# Low skeletal muscle mass as a proxy marker of sarcopenia is a risk factor for major complications in older patients undergoing curative colon resections for colon cancer

**DOI:** 10.3389/fmed.2024.1464978

**Published:** 2025-01-09

**Authors:** İsmail Tırnova, Maya Gasimova, Hatice Akay, Çağla Sarıtürk, Aslıhan Güven Mert, Özlem Yenidünya, Feza Yarbuğ Karakayalı

**Affiliations:** ^1^Department of General Surgery, Baskent University, Istanbul, Türkiye; ^2^Department of Radiology, Baskent University, Istanbul, Türkiye; ^3^Department of Statistics, Baskent University, Istanbul, Türkiye; ^4^Department of Internal Medicine, Division of Oncology Baskent University, Istanbul, Türkiye; ^5^Department of Anesthesiology, Baskent University, Istanbul, Türkiye

**Keywords:** colon cancer, sarcopenia, skeletal muscle mass index, geriatrics, complication, survival

## Abstract

**Introduction:**

Various reports have confirmed that low skeletal muscle mass, a proxy marker of sarcopenia, can be a risk factor for surgical and oncological outcomes in colon cancer. We aimed to investigate the effects of skeletal muscle mass index (SMMI) on postoperative complications, overall survival (OS), and disease-free survival (DFS) in older patients with colon cancer who underwent elective curative colon resections.

**Materials and methods:**

Patients over 65 years old with stage I-III colon cancer who underwent elective curative colon resections between January 2015 and December 2023 were included in this single-center retrospective longitudinal study. Demographics, comorbidities, laboratory data, pathological features, malignant lymph node ratio (MLNR), OS, and DFS were recorded. Controlling Nutritional Status (CONUT) Score was used to assess the nutritional status. An axial portal-phase image was obtained at the level of the third lumbar vertebra, and muscle areas were calculated. SMMI was calculated by dividing the muscle area (cm^2^) by the square of the patient’s height (m^2^). Low SMMI was defined as SMMI<41 cm^2^/m^2^ in women and < 43 cm^2^/m^2^ in men with body mass index (BMI) <25 kg/m^2^, and as SMMI <53 cm^2^/m^2^ in patients with a BMI >25 kg/m^2^. Postoperative complications were classified according to the Clavien-Dindo system. Univariate and multivariate analyses were performed to investigate the factors related to the postoperative complications, OS and DFS.

**Results:**

In total, 98 cases (mean age 75.2 ± 6.9, 55.1% male) were included in the study. The median follow-up time was 38.3 (0.5–113) months. There were 64 patients (65.3%) in the Low SMMI group and 34 patients (34.7%) in the Normal SMMI group. Logistic regression analysis demonstrated that low SMMI was associated with a higher risk of major complications, with an odds ratio of 5.3 (95% CI, 1.1–20.1; *p* = 0.037). Cox regression analysis revealed no significant differences in OS and DFS.

**Conclusion:**

Low SMMI as a proxy marker of sarcopenia was found to be an independent risk factor for postoperative major complications. Additional prospective studies are warranted to obtain more reliable results.

## Introduction

Colorectal cancer is the third most common cancer in the world with an incidence of more than 1.8 million new cases and more than 800,000 deaths in 2018 (1). With the expansion of the world population, decreasing non-cancer-related deaths, and the rise in life expectancy, as well as the increase in diagnostic tools such as colonoscopy and cross-sectional imaging, the incidence of colorectal cancer is increasing (2). Approximately 40% of colorectal cancer patients are older than 75 years and the incidence is presumed to grow in the future with the aging population (3). Although colon and rectal cancer are often examined together, the effectiveness of neoadjuvant chemoradiotherapy in the treatment of rectal cancer constitutes the main difference in treatment approaches. Neoadjuvant treatment strategies may have potentially negative effects on muscle mass in rectal cancer patients.

Sarcopenia is a progressive and generalized skeletal muscle disorder that is associated with an increased likelihood of adverse outcomes in both medical aspects and physical performances, especially for older patients (4). The European Working Group of Sarcopenia in Older People (EWGSOP) previously published their first report in 2010 to define sarcopenia clearly (5). In that report detected lower muscle mass was defined as presarcopenia. Lower muscle strength or lower physical performance was needed to establish the diagnosis of sarcopenia. In 2019 the group revised the definition of sarcopenia (EWGSOP2). Lower muscle strength was defined as probable sarcopenia. Lower muscle quantity or quality was needed to establish the diagnosis of sarcopenia. Severe sarcopenia was diagnosed in the presence of low physical performance in addition to the first two criteria (6).

There are various techniques to evaluate muscle strength during the diagnosis of sarcopenia. Handgrip strength and chair sit-to-stand tests are the most common methods that are suggested to be evaluated by EWGSOP2 (6). Although these tests are not routinely applied in surgical and oncological practices. Therefore, deficiencies may occur in the definitive diagnosis and assessment of sarcopenia, especially in the oncological patient groups, especially in retrospective studies. Muscle quantity measurements can be performed with various imaging techniques. Some complex methods such as Dual-energy X-ray absorptiometry (DXA) and Bioelectric Impedance Analysis (BIA) are used to measure muscle mass. However, it is easier to use computed tomography (CT) and magnetic resonance (MR) images already available during oncological evaluations of patients in diagnosis, staging, and follow-up periods. Obtaining these images retrospectively is important in terms of cost-effectiveness and to prevent additional radiation exposure for patients. The muscle quantity may be calculated as the total skeletal muscle area and psoas muscle area by the level of the third lumbar vertebra (7). Since SMI can easily be calculated using MR and CT images at the L3 vertebra level in oncology and surgical practice, it is often used as a proxy marker for sarcopenia in studies and is considered more convenient.

In the scenario of the aging general population, and the increasing number of diagnosed and treated cancers in older adults, it has become more crucial to identify, understand, and assess the sarcopenia in older adults with cancer (8). Decreasing muscle mass is related to decreasing immunity, wound healing, and increased risk of death (9). Multiple reports have addressed the remarkable association between sarcopenia and the poor outcomes of different gastrointestinal system cancers (10–12). The potential negative effects of sarcopenia on colorectal cancer treatment have been addressed in many current studies, but studies specifically addressing this issue in older patients are limited (2, 13, 14). Cancer itself causes muscle loss, and aging also leads to muscle loss. In older adults, the presence of various systemic issues can increase susceptibility to adverse events, making the potential negative effects of sarcopenia more pronounced.

In the present study, we aimed to evaluate the effects of the skeletal muscle mass index as a proxy marker of sarcopenia; calculated by using the total skeletal muscle area at the level of the third lumbar vertebra in preoperative staging CT scans, on the postoperative complications, overall and disease-free survival rates for elective colon resections performed with curative intent for stage I-III colon cancer in our center. We specifically focused on colon cancer patients and excluded rectal cancer patients to obtain more homogenous results and to prevent possible negative effects of the neoadjuvant treatments on preoperative muscle mass.

## Materials and methods

### Participants and parameters

All patients who underwent colon resections at Baskent University Istanbul Hospital between January 2015 and December 2023 were retrospectively analyzed. Among patients aged 65 years and older, cases who underwent elective colon resection with curative intent due to stage I-III colon adenocarcinoma were included in the study. Patients with any histopathologic diagnosis other than adenocarcinoma, patients with stage IV disease at initial diagnostic work-up or intraoperative exploration, patients with rectal cancer, and patients without available CT scans were excluded.

Demographics, the American Society of Anesthesiologists (ASA) Scores, smoking status, acetylsalicylic acid and anticoagulant usage, comorbidities such as; coronary artery disease (CAD), congestive heart failure (CHF), hypertension (HT), diabetes mellitus (DM), chronic renal failure (CRF), chronic obstructive pulmonary disease (COPD), history of previous any surgical intervention for abdomen were recorded. Body mass index (BMI) was calculated by dividing the weight of the patient (kg) by the square of the patient’s height (m^2^). Preoperative serum albumin levels, total cholesterol levels, platelet counts, and lymphocyte counts were recorded. The nutritional status of the patients was assessed using the Controlling Nutritional Status (CONUT) Score, which is calculated based on serum albumin levels, total cholesterol levels, and lymphocyte counts. A higher CONUT Score shows worse nutrition.

Duration of operation, intraoperative complications, and conversion to laparotomy rates were recorded as intraoperative data. Postoperative outcomes; intensive care unit (ICU) stay requirements, length of ICU stay, blood transfusion requirements, surgical site infections (SSI), anastomotic leakage (AL) rates, and re-operation rates, were noted. Overall postoperative complications were recorded and classified according to the Clavien-Dindo scoring system (15). The Clavien-Dindo scoring system classifies the postoperative complications from I to V. The severity of the complications increases from class I to class V. Class V complication means the death of the patient. Comprehensive Complication Index (CCI) scores were calculated by using the Clavien-Dindo scores (16). CCI scores evaluate the sum of complications in the postoperative period, as percentages per patient. The Clavien-Dindo score V; the death of the patient means a 100% score of CCI. Patients with a Clavien-Dindo score of III and above were considered with major complications (15). The factors affecting the development of major complications were evaluated.

Pathological investigations of the resections as T stage and N stage, tumors largest diameter lymphatic invasions, perineural invasions, vascular invasions, grade, and differentiation of tumors were also evaluated. Malignant lymph node ratios (MLNR) were calculated by dividing the malign lymph node (LN) counts by the total harvested LN counts.

The adjuvant chemotherapy administrations were recorded in postoperative terms. Mortality rates were assessed for 30-day, 1-year, and 5-year following primary surgical intervention. The presence of recurrences of the disease was recorded. Overall survival was based on the duration from the colon resection to the death or the last follow-up. Disease-free survival was based on the duration from the colon resection to the development of local recurrence and/or distant metastasis or last follow-up. The possible effects of Low SMMI on overall survival (OS) and disease-free survival (DFS) rates were analyzed.

### Measurement of the skeletal muscle mass index

**Figure 1:**
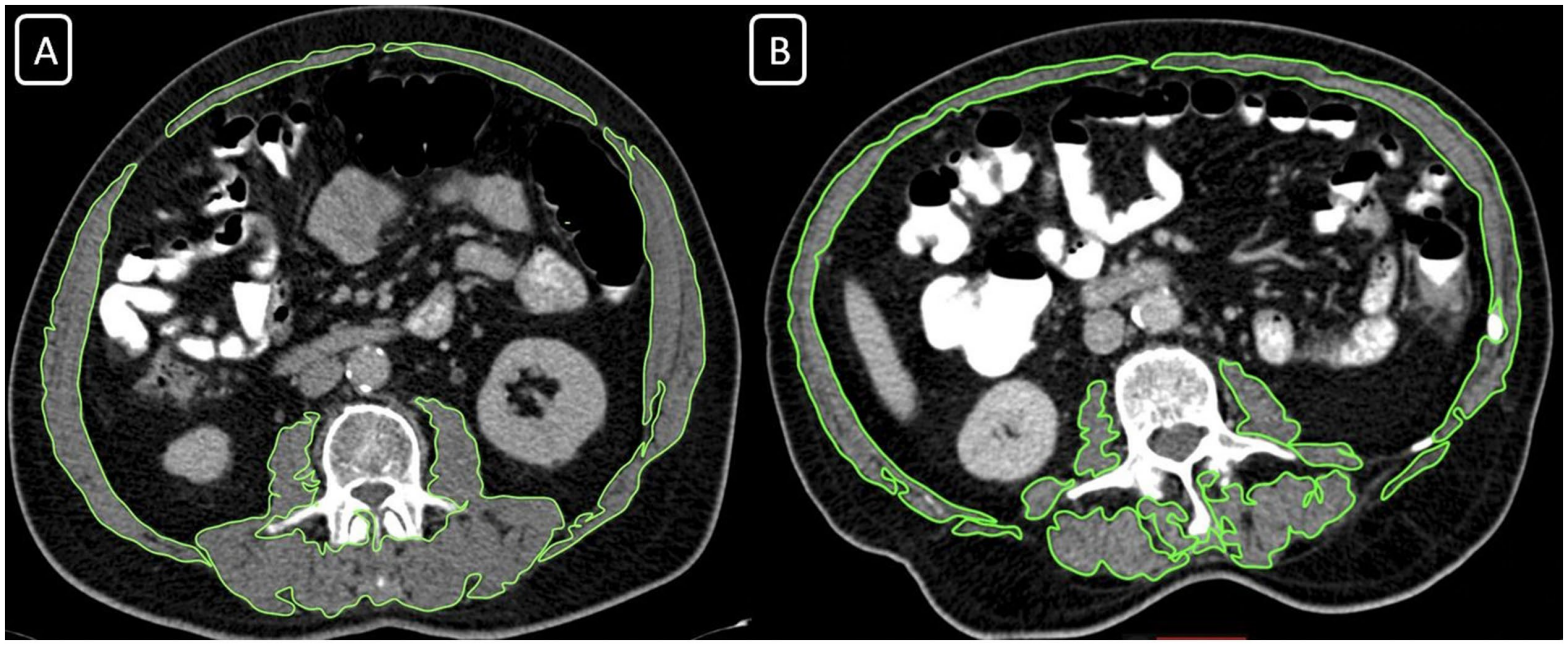
Computed tomography images at the L3 vertebral body, revealing the skeletal muscle area (SMA) measurement in two different individuals. **(A)** SMA = 150.33 cm^2^ with BMI: 24,7, normal skeletal muscle mass index group; **(B)** SMA = 150.33 cm^2^ with BMI: 22,4, low skeletal muscle mass index group.

The assessment of skeletal muscle mass (SMM) was performed by contrast-enhanced thoracoabdominal computed tomography (CT) for each patient as a routine evaluation for oncological staging (Siemens Somatom Sensation 64 Detector). For determining the SMM measurements; an axial portal-phase image was obtained at the level of the third lumbar (L3) vertebra. Two radiologists evaluated the images, one with over 15 years of academic experience and the other with 10 years of experience. The images were evaluated with commercial software (Osirix Lite). The software automatically scanned the images and, according to the specific densities of the different tissues, determined the areas with a density of −29 to 150 Hounsfield Units (HU) as muscle tissues (Figure 1). The radiologists checked, revised, and approved all muscle areas that the software program calculated. Then all muscle areas were measured as cm^2^. Skeletal muscle mass index (SMMI) was calculated by dividing the muscle area (cm^2^) by the square of the patient’s height (m^2^). We used the classification of Martin et al. to determine the groups for SMMI (17). Low SMMI was defined in women with SMI <41 cm^2^/m^2^ and men with SMMI <43 cm^2^/m^2^ in patients with body mass index (BMI) < 25 kg/m^2^. Low SMMI was defined in patients with >25 kg/m^2^ BMI level when their SMMI was lower than 53 cm^2^/m^2^ (17). The participants were classified into two groups; patients with Low SMMI, and Normal SMMI results.

### Ethical committee approval

This study was approved by the Baskent University Institutional Review Board (Project No: KA24/184–02.05.2024).

### Statistical analysis

Statistical analysis was performed using the statistical package *SPSS software* (Version 25.0, SPSS Inc., Chicago, IL, United States). If continuous variables were normally distributed, they were described as the mean ± standard deviation (*p* > 0.05) in the Kolmogorov–Smirnov test or Shapira-Wilk (*n* < 30). If the continuous variables were not normally distributed, they were described as the median. Comparisons between groups were evaluated using the Mann–Whitney U test. The categorical variables between groups were analyzed using the Chi-square test or Fisher’s Exact Test. A multivariate logistic regression analysis was performed to control for potential confounding factors and identify independent predictors of major complications. Predictor variables that were significant in the univariate analysis (*p* < 0.10) were included in the multivariate model. Kaplan–Meier survival curves were generated to evaluate the effect of preoperative skeletal muscle mass index on overall and disease-free survival rates. The log-rank test was used to compare survival distributions between groups. The association of the variables count with overall survival and disease-free survival were analyzed using the Cox proportional hazard model. A Cox regression model with stepwise selection was done to identify variables. The level of statistical significance was predetermined at *p* < 0.05 and %95 CI. Differences in the best overall response rates between the variables were analyzed with the Cochran–Mantel–Haenszel test.

## Results

**Figure 2:**
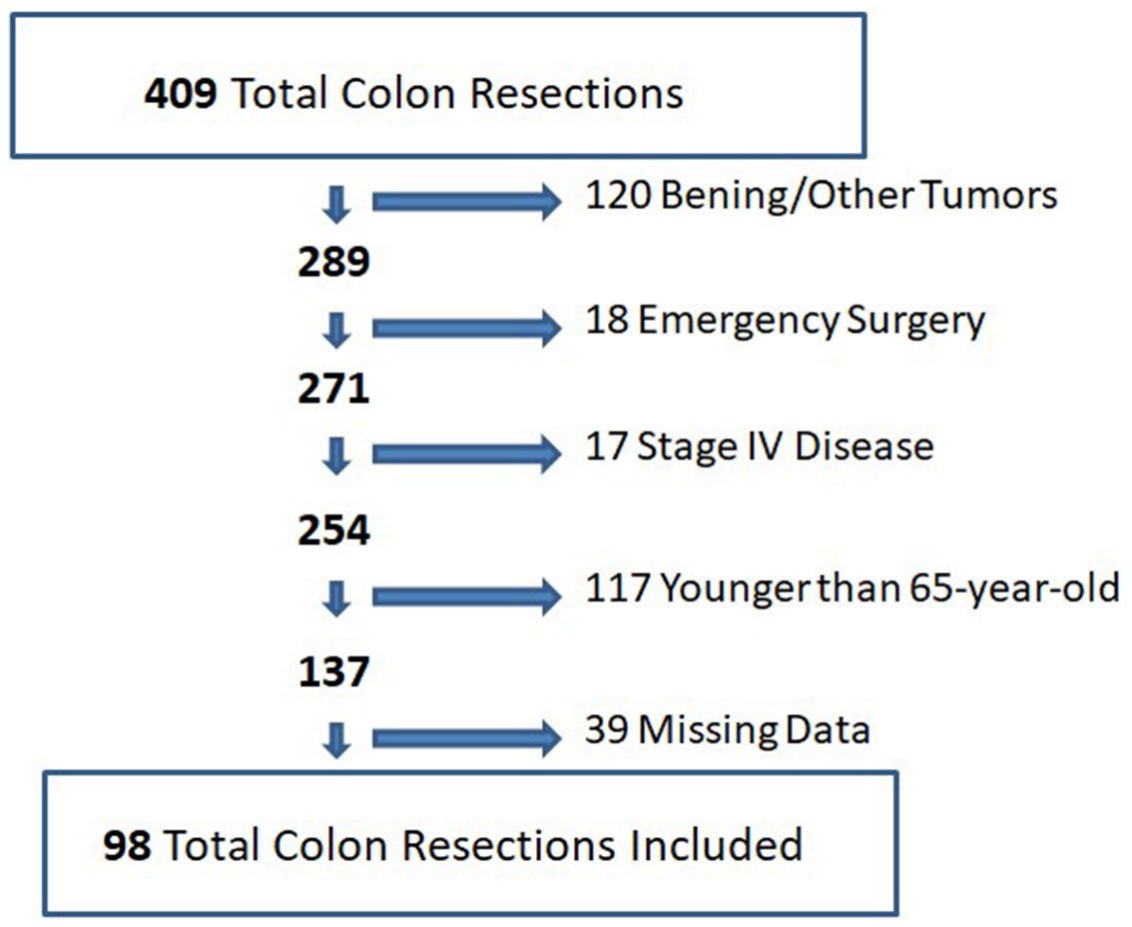
Flowchart diagram for included and excluded participants.

During the study period, a total of 409 consecutive colon resections were performed at our center. A total of 120 patients were excluded because the surgeries were for benign reasons (mostly for diverticular disease), 18 cases were excluded because they were emergency surgeries, 17 cases were excluded due to metastatic disease during the operation or preoperative work-up, 117 cases were excluded because the patients were under 65 years old, and 39 cases were excluded because of missing data. In total, 98 cases were included in the study (Figure 2).

The mean age of the cases was 75.2 (±6.9) years. There were 54 (55.1%) male and 44 (44.9%) female patients. The mean BMI was 25.5 (±4.4). The mean ASA score was 3. Twenty-four patients (24.5%) had a history of previous surgeries. In the Low SMMI group, there were 64 patients (65.3%), and in the Normal SMMI group, there were 34 patients (34.7%). The groups were similar in terms of demographic characteristics. The demographics and preoperative data of the patients are shown in Table 1.

**Table 1:** Demographics and the preoperative data of the participants stratified by skeletal muscle mass index.

Variable	Total *n* = 98	Skeletal muscle mass index (SMMI) classification		*p*-value
		Group low SMMI; (n;64/65.3%)	Group normal SMMI; (n;34/34.7%)	
Age	75.2 (±6.9)	75.3 (±6.8)	75.1 (±7.1)	0.862
Sex				0.525
Male Female	54 (55.1%) 44 (44.9%)	37 (57.8%) 37 (42.2%)	17 (50%) 17 (50%)	
Body-mass index (BMI)	25.5 (±4.4)	26.4 (±6.3)	25.1 (±2.9)	0.145
ASA score	3 (0–4)	3 (0–4)	3 (2–3)	0.164
Smoking (n,%)	13 (13.4%)	8 (12.7%)	5 (14.7%)	0.765
Acetyl salicylic acid usage (n,%)	42 (42.9%)	26 (40.6%)	16 (47.1%)	0.668
Anticoagulant usage (n,%)	15 (15.3%)	10 (15.6%)	5 (14.7%)	1
Coronary artery disease (n,%)	33 (33.7%)	20 (31.3%)	13 (38.2%)	0.508
Congestive heart failure (n,%)	14 (14.6%)	7 (11.3%)	7 (20.6%)	1
Hypertension (n,%)	74 (75.5%)	48 (75%)	26 (76.5%)	1
Diabetes mellitus (n,%)	36 (36.7%)	24 (37.5%)	12 (36.4)	1
Chronic renal failure (n,%)	2 (2.2%)	2 (3.2%)	0 (0%)	0.540
COPD (n,%)	5 (5.1%)	4 (6.3%)	1 (2.9%)	0.656
Previous surgical history (n,%)	24 (24.5%)	18 (28.1%)	6 (17.6%)	0.327
Plattelet (10^3^/μL)	260 (137–670)	292 (143–670)	240 (137–490)	0.085
Albumin (gr/dL)	3.7 (±0.5)	3.7 (±0,6)	3.8 (±0.5)	0.486
Lymphocyte (1/μL)	1931.6 (±816.3)	1804.8 (±867.7)	2170.3 (±816.3)	0.034*
Total cholesterol (mg/dL)	184.3 (±37.8)	182.9 (±41)	184.9 (±36.3)	0.806
CONUT score	1 (0–8)	1.5 (0–8)	1 (0–5)	0.191

SMMI, Skeletal muscle mass index; ASA, American Society of Anesthesiologists; COPD, Chronic obstructive pulmonary disease; CONUT, Controlling Nutritional Status; Mean: ±SD, Median: (min-max).

The asterisk (*) denotes values that are statistically significant.

The bold results indicate values that are statistically significant.

The mean surgery duration was 200.8 (±50.7) minutes. All cases were initiated laparoscopically. Sixteen patients (16.3%) were converted to open surgery. Four patients were converted due to mesenteric vascular bleeding, two due to inadequate surgical exposure, and 10 due to tumor invasion. Intraoperative complications occurred in six patients. These included splenic injury in one patient, small bowel injury in two patients, and mesenteric vein bleeding in three patients. In the LSMMI group, the left-sided tumor rates were higher (*p* = 0.019).

### Short-term surgical outcomes and complications

Twenty-nine patients required ICU stay following surgery. Twenty one patients (32.8%) from the Low SSMI group, and 8 patients (24.2%) from the Normal SMMI group stayed 0–7 and 0–10 days in ICU consecutively. There were no significant differences between the groups in terms of ICU requirement rates or ICU stay durations (Table 2).

**Table 2:** Operative and postoperative data of the participants stratified by skeletal muscle mass index.

Variable	Total *n* = 98	Skeletal muscle mass index (SMMI) groups		*P*-value
		Group low SMMI; (n;64/65.3%)	Group normal SMMI; (n;34/34.7%)	
Duration (Minutes)	200.8 (±50.7)	202.7 (±54.2)	197.2 (±43.9)	0.615
Intraoperative complication (n,%)	6 (6.1%)	5 (7.8%)	1 (2.9%)	0.145
Conversion to laparotomy (n,%)	16 (16.3%)	13 (20.3%)	3 (8.8%)	0.165
Tumor location				0.019*
Right Left	49 (50%) 49 (50%)	26 (40.6%) 38 (59.4%)	23 (67.6%) 11 (32.4%)	
ICU requirement (n,%)	29 (29.9%)	21 (32.8%)	8 (24.2%)	0.485
ICU duration (Days)	0 (0–10)	0 (0–7)	0 (0–10)	0.359
Postoperative blood transfusion	17 (17.3%)	13 (20.3%)	4 (11.8%)	0.403
Surgical site infection (n,%)	20 (20.4%)	15 (23.4%)	5 (14.7%)	0.431
Anastomotic leakage (n,%)	3 (3.1%)	3 (4.7%)	0 (0%)	0.549
Re-operation (n,%)	5 (5.1%)	5 (7.8%)	0 (0%)	0.160
Overall complications (n,%)	41 (41.8%)	29 (45.3%)	12 (35.3%)	0.394
Major complications Clavien-Dindo III Clavien-Dindo IV Clavien-Dindo V	23 (23.5%) 17 (17.3%) 5 (5.1%) 1 (1%)	19 (29.7%) 14 (21.8%) 5 (7.8%) 0 (0%)	4 (11.8%) 3 (8.8%) 0 (0%) 1 (2.9%)	0.049*
CCI (%)	13 (±20.7)	14.7 (±20.2)	9.9 (±21.6)	0.227
Adjuvant chemotherapy (n,%)	58 (59.2%)	37 (57.8)	21 (61.8%)	0.830
Mortality (n,%)				
30-days 1-year 5-year	1 (1.1%) 6 (6.1%) 29 (29.6%)	0 (0%) 5 (7.8%) 21 (32.8%)	1 (2.9%) 1 (2.9%) 8 (23.5%)	1 0.661 0.365

SMMI, Skeletal muscle mass index; ICU, Intensive Care Unit; CCI, Comprehensive Complication Index Mean: ±SD, Median: (min-max).

The asterisk (*) denotes values that are statistically significant.

The bold results indicate values that are statistically significant.

Complications were developed in 41 patients (41.8%). Twenty nine patients (45.3%) from the Low SMMI group and 12 patients (35.3%) from the Normal SMMI group suffered postoperative complications (*p* = 0.394). The mean CCI value was 13 (±20.7). The Low SMMI group’s CCI was 14.7 (±20.2), and the Normal SMMI group’s CCI was 9.9 (±21.6) (*p* = 0.227). Major complications developed in 23 patients (23.5%). The rate of major complications was statistically higher in the Low SMMI group; 19 patients (29.7%) and 4 patients (11.8%) respectively (*p* = 0.049). Seventeen patients (17.3%) required postoperative transfusion. The groups were similar for transfusion rates (*p* = 0.403). No patient required re-operation due to bleeding. The groups were similar for re-operation rates (*p* = 0.160). Surgical site infection (SSI) was detected in 20 patients (20.4%). Four patients required vacuum-assisted closure (VAC) therapy for SSI. The groups were similar for SSI (*p* = 0.431). The rates of adjuvant chemotherapy were similar in both groups. Pathological investigations revealed similar outcomes (Table 3).

**Table 3:** Pathological findings of the tumors, and differences between low and normal skeletal muscle mass index groups.

Variable	Total *n* = 98	Skeletal muscle mass index (SMMI) groups		*P*-value
		Group low SMMI; (n;64/65.3%)	Group normal SMMI; (n;34/34.7%)	
Tumor largest diameter (mm)	50 (10–100)	45 (10–100)	50 (10–100)	0.132
T stage				0.638
I II III IV	2 (2%) 12 (12.2%) 56 (57.1%) 28 (28.6%)	1 (1.6%) 9 (14.1%) 34 (53.1%) 20 (31.3%)	1 (2.9%) 3 (8.8%) 22 (64.7%) 8 (25.5%)	
N stage				0.873
0 I II	64 (65.3%) 19 (19.4%) 15 (15.3%)	42 (65.6%) 13 (20.3%) 9 (14.1%)	22 (64.7%) 6 (17.6%) 6 (17.6%)	
TNM stage				0.937
I II III	13 (13.3%) 52 (53.1%) 33 (33.7%)	9 (14.1%) 34 (53.1%) 21 (32.8%)	4 (11.8%) 18 (52.9%) 12 (35.3%)	
Tumor grade				0.506
I II III	44 (44.9%) 34 (34.7%) 20 (20.4%)	28 (45.3%) 24 (37.5%) 11 (17.2%)	15 (44.1%) 10 (29.4%) 9 (26.5%)	
Tumoral differentiation				0.553
Well Moderate Poor	38 (38.8%) 40 (40.8%) 20 (20.4%)	26 (40.6%) 27 (42.2%) 11 (17.2%)	12 (35.2%) 13 (38.2%) 9 (26.5%)	
Lymphatic invasion				0.193
No Yes	37 (37.8%) 61 (62.8%)	21 (32.8%) 43 (67.2%)	16 (47.1%) 18 (52.9%)	
Vascular invasion				1
No Yes	75 (76.5%) 23 (23.5%)	49 (76.6%) 15 (23.4%)	26 (76.5%) 8 (23.5%)	
Perineural invasion				0.245
No Yes	70 (71.4%) 28 (28.6%)	43 (67.2%) 21 (32.8%)	27 (79.4%) 7 (20.6%)	
Harvested lymph nodes count	22 (8–62)	22.5 (8–40)	22 (10–62)	0.132
Malignant lymph nodes count	0 (0–32)	0 (0–8)	0 (0–32)	0.288
MLNR (%)	0 (0–92)	0 (0–40)	0 (0–92)	0.333
Metastasis/Recurrence				0.825
Liver Lung Peritoneal	18 (18.4%) 9 (9.2%) 9 (9.2%)	11 (17.1%) 5 (9.1%) 8 (12.5%)	7 (20.6%) 4 (11.8%) 1 (2.9%)	

SMMI, Skeletal muscle mass index; MLNR, Malignant Lymph Node Ratio; Mean: ±SD, Median: (min-max).

The factors related to the major complications were evaluated (Table 4). Higher ASA score, higher CONUT score, presence of congestive heart failure, and presence of Low SMMI were related to developing postoperative major complications in univariate analysis. In addition to these factors, age, and sex distribution were evaluated with a multivariate logistic regression model. Besides Low SMMI (OR: 5.3, 95% CI: 1.1–20.1, *p* = 0.037), higher ASA scores (OR: 2.9, 95% CI: 1.1–8.7, *p* = 0.019), and the presence of congestive heart failure (OR: 4.1, 95% CI: 1.1–15.4, *p* = 0.036) were identified as risk factors for developing major complications (Table 4).

**Table 4:** Factors related to major complications.

Variable	Univariate analysis			Multivariate analysis		*P*-value
	Major complication		*P*-value			
	Yes (*n* = 23/23.5%)	No (*n* = 75/76.5%)				
				Odds ratio	(95% CI)	
Age	75.6 (±4.4)	74.9 (±7.6)	0.592	0.98	(0.9–1.1)	0.649
Sex			1	1.08	(0.36–3.2)	0.887
Male	13 (56.5%)	41 (54.7%)				
Female	10 (43.5%)	34 (45.3%)				
ASA score	3 (2–4)	2 (0–3)	***0.019***	***2.9***	(1,1–8.7)	***0.050***
CONUT score	3 (0–8)	1 (0–8)	***0.037***	1.1	(0.85–1.42)	0.474
Congestive heart failure	7 (30.4%)	7 (9.6%)	***0.036***	***4.1***	(1,1–15.4)	***0.026***
Low SMMI	19 (82.6%)	45 (60%)	***0.037***	***5.3***	(1,1–20.1)	***0.013***

CI, Confidence Interval; ASA, American Society of Anesthesiologists; CONUT, Controlling Nutritional Status; NS, Nonsignificant; SMMI, Skeletal muscle mass index. Logistic regression analysis.

The bold results indicate values that are statistically significant.

### Overall survival and disease-free survival outcomes

Median follow-up time was 38.3 (0.5–113) months. The factors related to the OS rates were age, perineural invasion, presence of Low SMMI, malignant lymph node ratio (MLNR), and CONUT score in univariate analysis. The Kaplan–Meier survival analysis is shown in Table 5. Our analysis showed that Low SMMI is related to worse OS rates (*p* = 0.044). Besides the factors in the univariate analysis, sex was included in the multivariate analysis. Multivariate Cox regression analysis revealed that advanced age, the presence of perineural invasion, higher MLNR, and higher CONUT scores were risk factors for worse OS rates (Table 6).

**Table 5:** Kaplan–Meier survival analysis for overall and disease-free survival.

Variable	n	Estimate mean	Std. Error	95%CI		1-year survivor %	3-year survivor %	5-year survivor %	*P*-value
				Lower bound	Upper bound				
Overall survival (months)	98	78.7	4.9	68.9	88.5	92.6	74.3	60.0	***0.044***
LSMMI	64	68.9	6.9	55.4	82.6	90.2	68.1	50.1	
NSMMI	34	86.1	6.3	73.8	98.4	97.1	84.1	73.1	
Disease-free survival (months)	98	77.8	5.1	67.9	87.7	87.0	71.1	60.4	0.631
LSMMI	64	76.2	6.6	63.3	89.0	79.9	67.5	61.4	
NSMMI	34	71.9	6.5	59.1	84.7	89.4	77.1	60.5	

LSMMI, Low skeletal muscle mass index; NSMMI, Normal skeletal muscle mass index.

The bold results indicate values that are statistically significant.

**Table 6:** Factors related to overall survival.

Variable	Univariate analysis			Multivariate analysis		
	Hazard Ratio	(95% CI)	*P*-value	Hazard ratio	(95% CI)	*P*-value
Age	1.1	(1.01–1.2)	***0.030***	***1.1***	(1.01–1.3)	***0.015***
Sex, Male	1	(0.5–2.1)	0.940	1.4	(0.63–3.29)	0.379
Lymphatic invasion	1.8	(0.8–3.9)	0.151	2.21	(0.79–6.25)	0.132
Vascular invasion	1.4	(0.7–3.1)	0.342	1.62	(0.64–4.12)	0.306
Perineural invasion	2.3	(1.1–4.9)	***0.027***	***2.9***	(1.3–6.3)	***0.009***
LSMMI	2.2	(1.01–5)	***0.044***	1.16	(0.47–2.83)	0.742
MLNR	1.1	(1.1–1.2)	***0.002***	***1.1***	(1.01–1.2)	***0.0001***
CONUT score	1.2	(1.1–1.4)	***0.006***	***1.2***	(1.1–1.5)	***0.006***

CI, Confidence Interval; LSMMI, Low skeletal muscle mass index; MLNR, Malignant Lymph Node Ratio; CONUT, Controlling Nutritional Status. Cox regression analysis.

The bold results indicate values that are statistically significant.

**Figure 3:**
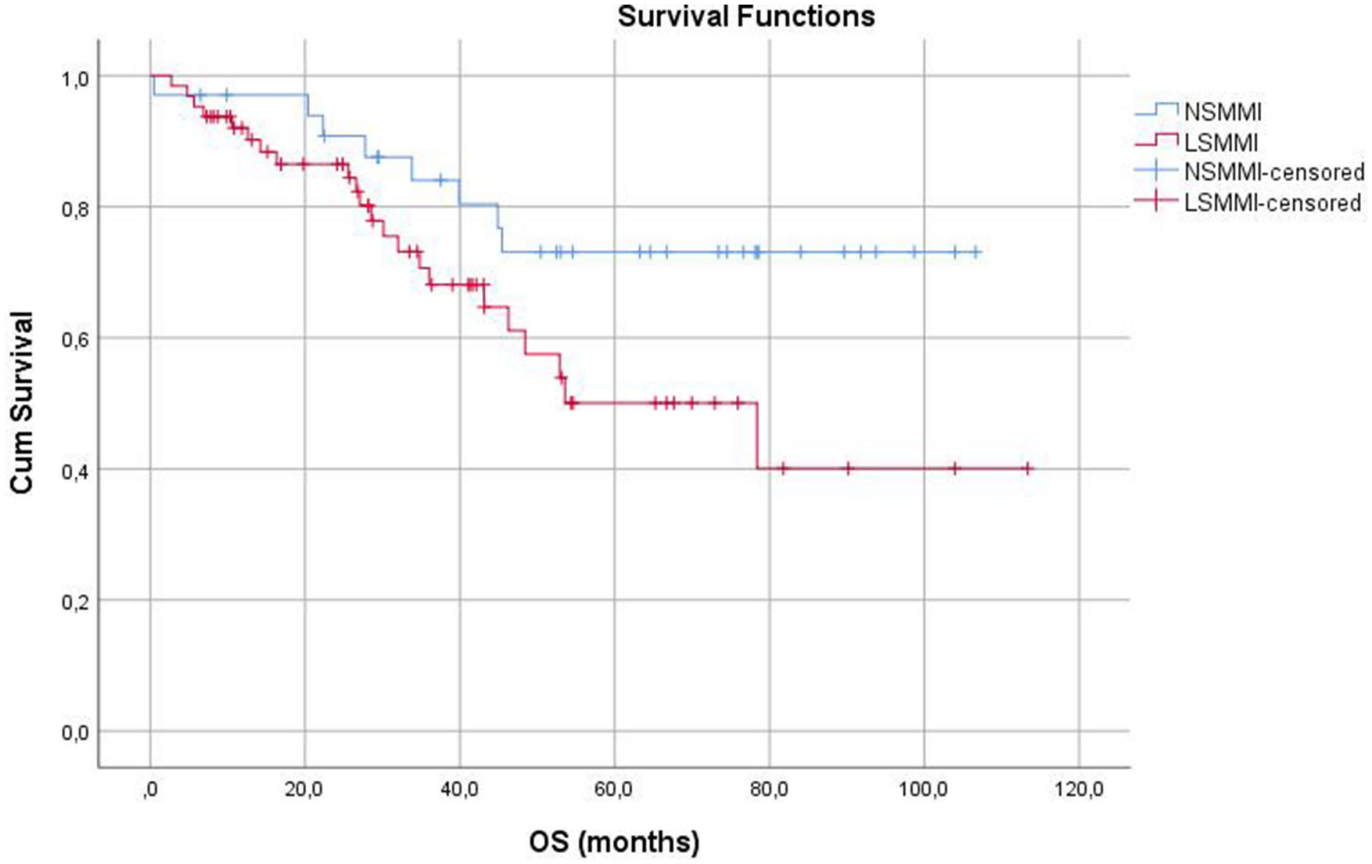
Kaplan–Meier graphics for overall survival (months).

**Figure 4:**
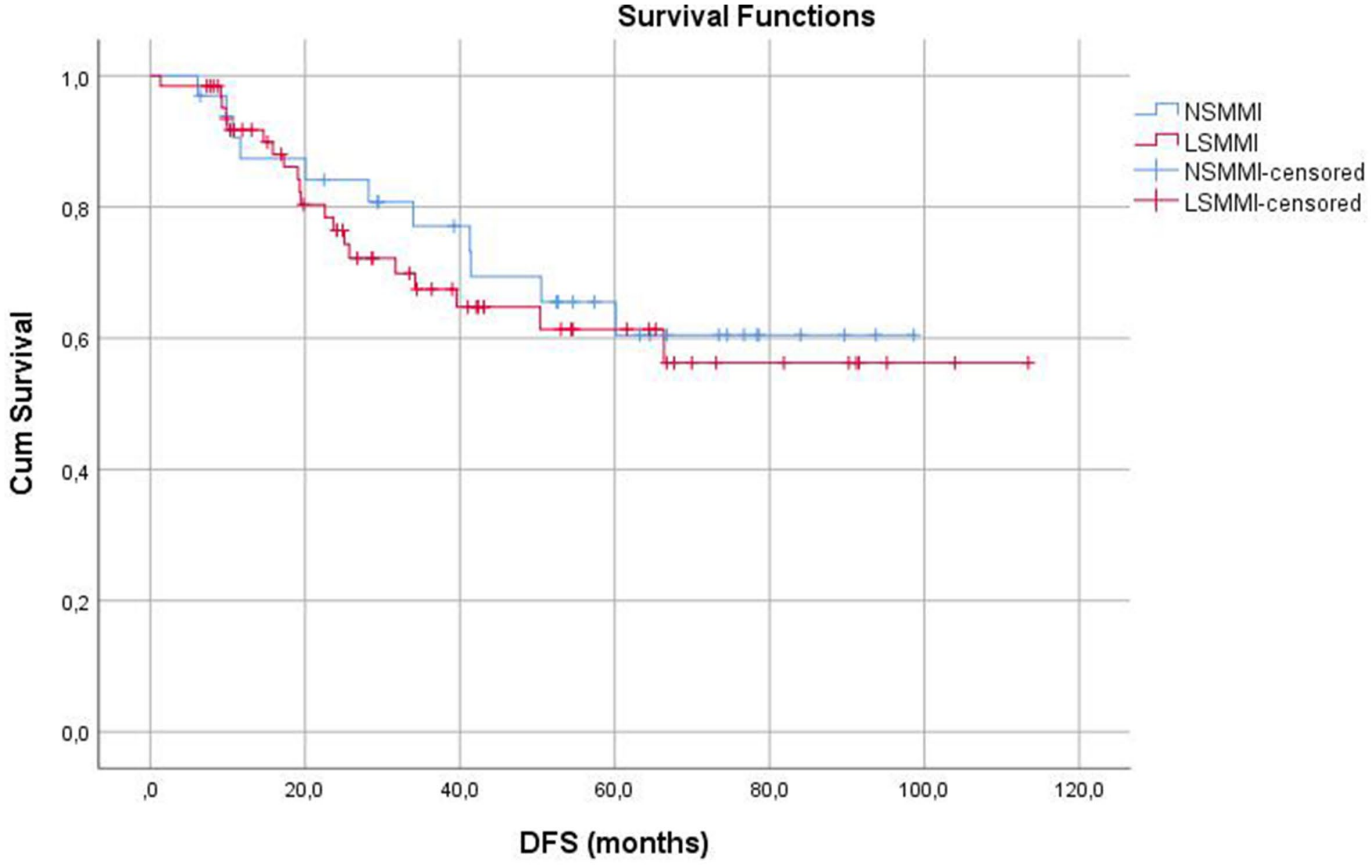
Kaplan–Meier graphics for disease-free survival (months).

There were no differences between the groups for DFS rates. The Kaplan–Meier graphics comparing the groups for Overall Survival (OS) and Disease-Free Survival (DFS) were shown in Figures 3, 4, respectively.

## Discussion

This single-center, longitudinal retrospective study revealed that low skeletal muscle mass index, as a proxy marker of sarcopenia, was an independent risk factor for developing major postoperative complications in older patients undergoing elective, curative colon resection for stage I-III colon cancer (OR: 5.3) (Table 4). Secondly, although our study could not demonstrate low SMMI as a risk factor for overall survival, advanced age, the presence of perineural invasion, higher MLNR, and higher CONUT scores were shown to be independent risk factors for poor overall survival (Table 6).

Although it requires muscle strength measurements to diagnose sarcopenia, low muscle mass or mass indexes are usually considered as proxy markers of sarcopenia in surgery and oncology practice. The effects of low muscle mass and sarcopenia on colorectal cancer are also widely analyzed. Consistent with our outcomes, low skeletal muscle mass is predictive of postoperative complications in patients undergoing oncological colorectal surgery in other studies (6, 18–22). Although there were no significant differences, the overall complications were slightly higher in the Low SMMI group in our study (45.3% vs. 35.3%, *p* = 0.394). Overall complication rates have been reported as 32–61% in the literature (21, 22). Our overall complication rate of 41.8% was comparable with current data. The majority of the publications in the literature reported significantly higher overall complication rates in low muscle mass groups (21–25). Xiao et al. enrolled 1,630 patients (mean age 64 ± 11.3) who underwent colon resections for stage I-III colon cancer in their study. The multivariate analyses showed that low L3 vertebra skeletal muscle mass index was a risk factor for postoperative overall complications (21). Chai et al. enrolled 228 patients (median age 69) who underwent colorectal resections due to colorectal cancer. They showed that L3 vertebra low SMI was a risk factor for postoperative overall complications (22). Oh et al. enrolled 423 patients who underwent colon resections due to colon cancer and showed that L3 vertebra low SMI was a risk factor for postoperative overall complications (23). Jochum et al. enrolled the patients who underwent rectum resections for diagnosis of rectal cancer and their multivariate analyses showed that L3 vertebra low SMI was a risk factor for postoperative overall complications (24). Richards et al. enrolled 350 colorectal cancer patients who underwent colorectal resections and used psoas muscle index (PMI) calculated by CT scans and used as a proxy marker of sarcopenia. Their study revealed that low PMI was a risk factor for postoperative overall complications (25). Although previous studies have mostly shown that low SMI is associated with increased postoperative complications (21–25), there are also studies where this association could not be demonstrated (26). Aro et al. enrolled 348 colorectal cancer patients to their study (26). They determined the sarcopenia via SMMI in the CT scans and used the cut-off values described by Martin et al. (17). They reported similar overall complication rates in both low and normal L3 vertebra skeletal muscle mass index groups (26). The CCI score is a useful tool to demonstrate overall complications. The mean CCI rate for our Low SMMI group was 14.7. The Normal SMMI group had 9.9 mean CCI and the difference was not statistically significant (*p* = 0.227). Batista et al. enrolled 639 colorectal resections in their study (27). They analyzed both benign and malignant colorectal resections and determined the sarcopenia via psoas muscle index in the CT scans, by dividing the bilateral psoas area by patient height^2^ (27). Their low PMI group’s CCI score was 16.1% higher when compared to the normal PMI group, and it was statistically significant (27). The fact that only the older patient group was enrolled in our study and that the number of patients was relatively small compared to the literature examples above may have caused us not to detect a significant difference in overall complications. This highlights the need for further studies, particularly in the older population, in this area.

Major complication (Clavien-Dindo≥III) rates have been reported to be 16–32% in the literature (18, 22, 23, 25). Our major complication rate was 23.5%. This was comparable with current data. Trejo-Avila et al. have shown that sarcopenia (criteria for sarcopenia was limited to SMM assessment or SMM plus physical performance or strength) is associated with increased major complications after colorectal cancer resections with 4,514 patients included meta-analysis (18). Chai et al. defined sarcopenia by SMMI, which was calculated by CT images obtained at the L3 vertebra level, and reported low muscle mass index as an independent risk factor for developing major complications after colorectal cancer resections (22). Richards et al. and Batista et al. defined sarcopenia by the psoas muscle index in colorectal cancer patients, and both of them prompted sarcopenia as an independent risk factor for developing major complications in the postoperative period (25, 27). In contrast, Aro et al. reported similar major complication rates in low and normal muscle mass groups (26).

Only three anastomotic leakage (AL) were detected in our patients (3.1%). All three were in the Low SMMI group but it wasn’t statistically significant. Our AL rates were correlated with the study by Richards et al. published (25). Oh et al. reported 1.4% of AL rates and all 5 patients with AL were in the normal SMI group (23). Fleming et al. and Chai et al. reported 7.2% rates of AL (22, 28). Their results also showed no statistical significance between the groups. A large sample-size meta-analysis has shown similar AL rates between low and normal SMI groups (18). Li et al. reported a higher risk for AL for colorectal resections in low PMI patients (29). The lower rates of AL in elective colorectal surgery may lead to this lack of significance in statistical analyses.

Preoperative detected low muscle mass index was identified as an independent risk factor for overall survival (OS) rates after colorectal cancer resections in multiple studies (6, 30–32). Golder et al. revealed worse overall survival rates for the low muscle mass group in an 1,146 patients-included study. Low muscle mass was defined by CT images at the L3 vertebra level (31). A large sample-size (*n* = 728) study published by Shirdel et al. from Sweden, showed statistically significant shorter OS rates for the low muscle mass group among stage I-III colorectal cancer patients (33). Hopkins et al. included 968 colorectal cancer patients and defined sarcopenia with SMMI, which was calculated via CT images at the L3 vertebra level. Hopkins et al. revealed worse overall survival rates in their low SMMI group (32). Oh et al. enrolled 423 patients with stage I-III colon cancer in their study, and revealed similar overall survival rates in low and normal skeletal muscle mass groups which were classified according to the SMMI (23). Black et al. analyzed 339 colorectal cancer patients and evaluated the SMMI. Their results showed that the low SMMI group had worse OS rates in univariate analysis. On the other hand, the multivariate Cox regression analysis they performed, could not mark low SMMI as an independent risk factor for OS, similar to our results (14). Differences in study designs, the number of participants, tumor locations (colon, rectum, colorectal), and follow-up times may lead to heterogeneous outcomes.

Disease-free survival rates were similar between the Low SMMI and Normal SMMI groups in our study. On the other hand, Sueda et al., Hopkins et al., Trejo-Avila et al., and Ojima et al. have shown worse DFS for low SMMI groups (2, 18, 32, 34). The fact that we had a relatively small number of patients may be the reason why we did not find a significant difference in terms of DFS.

The majority of the publications enrolled elective patients in their analyses, but Lee et al. investigated the prognostic role of pre-sarcopenia (defined as low skeletal muscle mass) on obstructive colorectal cancer patients (35). Interestingly their results showed similar outcomes compared with the rest of the literature. The postoperative complication rates were similar in both groups. Moreover, the overall survival rates were shorter in the low SMMI group.

In the present study, the mean ages of Low SMMI and Normal SMMI groups were very similar; 75.3 and 75.1 consecutively. Limited articles are evaluating the relationship between muscle mass and outcomes of colon resections specifically in older colon cancer patients. Ojima et al. included 142, and Looijaard et al. included 284 geriatric colon cancer patients in their trials (2, 36). Ojima et al. studied the effects of psoas muscle mass index (PMI) and intramuscular adipose tissue content (IMAC) in patients with colon cancer. The mean age of the patient group in their study was 80.9 years and low PMI and high IMAC were associated with worse overall survival (2). Looijaard et al. examined a group of colon cancer patients with a mean age of 73.9 years and showed the relationship between muscle mass and density and postoperative major complications (36). These two articles have a median age similar to our participants. Most of the literature is composed of lower-aged participants. Moreover, in publications examining the relationship between colorectal cancer and low skeletal muscle mass as a proxy marker of sarcopenia, it can be seen that the low muscle mass patient group is older, even if it is not statistically significant (13, 21, 22, 24–27). The incidence of low muscle mass is higher in the older age group, and we think that the similar age distribution of the two groups in our study ensures that the effect of the age variable does not affect the results (13, 37). On the other hand, in similar studies involving both younger adults and geriatric patients, the ratio of the low muscle mass group to all patients varied between 15 and 54%, while this ratio was found to be 65% in our study (21, 23, 34). This was the main feature that distinguished our current study from other Western literature samples.

Additionally, the mean BMI levels are also similar between the groups. There were 3 obese patients in Low SMMI group and 8 obese patients in Normal SMMI group. The nutritional parameters such as albumin levels and CONUT scores, and oncological status such as TNM stages, and tumoral grades are also similar and this provides us a more reliable comparison between the two groups. The median number of harvested lymph nodes was found to be 22. This result can be considered as an indicator that adequate oncological evaluation could be performed in our patients. On the other hand, the fact that DFS rates are similar in both groups is also remarkable in that it suggests that the poor results in the Low SMMI group in the OS rates are not due to cancer progression but to the general medical condition of the patients. Although the exclusion of rectal cancer patients, for whom the neoadjuvant approach has an important place in the treatment strategy, causes a decrease in the number of patients, we think that it adds importance to our study in terms of data homogenization. Additionally, the proportion of patients receiving adjuvant chemotherapy was similar in the two groups.

One of the interesting differences in our groups was the tumor locations. The low SMMI group consisted of mostly left-sided tumors. The normal SMMI group consisted of mostly right-sided tumors. It is generally thought that right-sided colon tumors become symptomatic in more advanced stages and the negative effects of cancer on patients will be more profound, especially in nutritional manner and loss of muscle mass. However, there is no convincing evidence in the literature. On the other side, some studies show no relation between the tumor locations and the disease durations (38, 39). Öztürk et al. revealed no statistically significant correlation between the duration of symptoms and either tumor site or stage, in their large sample-size prospective, multi-center study from Turkiye (38). Young et al. also revealed no relation between tumor location and delayed diagnosis of colon cancer. There is no consensus in the medical literature on this issue. Future studies may provide more reliable results in this regard.

We have some considerable limitations for this trial. The retrospective design of the trial is the main limitation itself. Due to the retrospective design, we focused on muscle mass measurements and could not assess grip strength, the key parameter for sarcopenia. On the other hand, as we evaluated only colon cancer patients, a limited number of patients could be included in the present study. Prospective and multicentric trials with larger sample sizes may provide more realistic results to be achieved. Moreover, a considerable portion of stage I-III colon cancer patients require adjuvant chemotherapy. The effects of adjuvant therapies may cause alteration both positively and negatively for therapeutic processes, complications, and outcomes. Future studies may also include this variable in statistical analysis. Additionally, using skeletal muscle quality and density parameters, which are also used in the evaluation of sarcopenia, may provide more objective results.

## Conclusion

The low skeletal muscle mass was highly prevalent in older patients with colon cancer and associated with increased major complications in the present study. We do not know whether effective interventions in patients with low muscle mass affect surgical and oncological outcomes; this is an area that requires further research. Therefore, we suggest screening for sarcopenia in older patients who are candidates for curative resection for colon cancer, and effective interventions for sarcopenia should be further explored through prospective interventional studies to improve outcomes and may serve as a tool for clinicians in this particular group of patients. Additional prospective studies analyzing large sample sizes, and integration of the muscle strength tests for screening the sarcopenia for the older patients are warranted to obtain more reliable results for revealing the effects of sarcopenia on outcomes of colon cancer therapy.

## Data Availability

The raw data supporting the conclusions of this article will be made available by the authors, without undue reservation.
